# A systematic review of qualitative research exploring patient and health professional perspectives of breakthrough cancer pain

**DOI:** 10.1007/s00520-023-08076-9

**Published:** 2023-12-13

**Authors:** G. B. Crawford, A. Lakhani, L. Palmer, M. Sebalj, P. Rolan

**Affiliations:** 1 Faculty of Health & Medical Sciences, University of Adelaide, Adelaide Medical School, North Terrace, Adelaide, SA 5000 Australia; 2 Northern Adelaide Palliative Service, Northern Adelaide Local Health Network, Modbury Hospital, 41-69 Smart Road, Modbury, SA 5092 Australia; 3 The School of Psychology and Public Health, La Trobe University, 360 Collins St, Melbourne, VIC 3000 Australia; 4 The Hopkins Centre, Menzies Health Institute Queensland, Griffith University, University Drive, Logan CampusMeadowbrook, QLD 4131 Australia; 5 Palliative Care Department, Eastern Health, 251 Mountain Highway, Wantirna, VIC 3152 Australia; 6 Northern Adelaide Pain Service, Northern Adelaide Local Health Network, Modbury Hospital, 41-69 Smart Road, Modbury, SA 5092 Australia

**Keywords:** Breakthrough cancer pain, Palliative care, Health professional, Patients, Qualitative, Systematic review

## Abstract

**Purpose:**

Breakthrough cancer pain (BtCP) is a prevalent health issue which is difficult to manage. A plethora of quantitative research in this area exists. There is a paucity of research on the perspectives of health professionals and patients surrounding domains impacting effective treatment, including definitions of BtCP, treatment, and education opportunities. This review aims to identify and synthesize the extent of qualitative research exploring health professional and patient perspectives of BtCP.

**Methods:**

A systematic review following the Preferred Reporting Items for Systematic Reviews and Meta-Analyses (PRISMA) approach was undertaken. The approach was registered with Prospero. MEDLINE, EMBASE, and Web of Science were searched for peer-reviewed literature published any date prior to May 19, 2022. Eligible sources must have considered health professional and/or patient perspectives of BtCP. A narrative synthesis approach was utilized.

**Results:**

Three sources met the review criteria. One source explored nurse perspectives, while two sources explored patient perspectives. Study quality was moderate to high. Overlapping themes across the three studies included communication, defining BtCP, impact of BtCP, management of BtCP, perceptions of BtCP, analgesia and pain relief, and training and professional development.

**Conclusion:**

Given limited research investigating clinician and patient perspectives of BtCP, a rich understanding informed by exploratory qualitative methods around identification, best management strategies, professional development, and factors promoting and inhibiting best practice remains unclear. Further qualitative inquiry is warranted, and it is expected such research will inform BtCP clinical guidelines.

## Introduction

Breakthrough cancer pain (BtCP) is defined as an episode of severe transient pain in patients with cancer [1, 2]. It “breaks through” persistent background pain which is usually managed by opioids [1, 2]. BtCP can affect mobility and quality of life [1] and is a factor which contributes to patient morbidity [3]. It incurs considerable healthcare costs, with yearly pain-related hospitalizations costing five times more for BtCP patients compared to cancer patients without BtCP [4]. BtCP is common [3, 5] and is difficult to diagnose and treat [5, 6]. A systematic review of 19 high-quality articles found the pooled prevalence of BtCP was 59% in patients with mixed cancer diagnoses; prevalence estimates were varied with the lowest being 40% and the highest being 81% [7]. Canal-Sotelo et al. [6] found differences in estimates could be partially explained by differing definitions of BtCP, as well as differences in prevalence across patient settings (e.g., inpatient versus outpatient clinics). This view is supported by Vellucci et al. [8], who confirm that the lack of a clinical consensus around what constitutes BtCP and universally accepted diagnostic tools, in addition to an underestimation of severity, contribute to the variability in prevalence rates, and hinder clinical management.

Management of BtCP can involve pharmacological and non-pharmacological methods [3]. The management process of BtCP typically involves assessment, treatment, reassessment, and follow-up [3]. The most common pharmacological method to manage BtCP is opioid medication [2]. The pharmacological management of BtCP with opioid medication (such as morphine) is recommended by the World Health Organization [WHO] [9] (see WHO Guidelines for the pharmacological and radiotherapeutic management of cancer pain in adults and adolescents [8]). A meta-analysis investigating the efficacy of opioid analgesics for BtCP found that all BtCP medications had an effect on pain relief, with transmucosal fentanyl medications providing effective relief in a shorter timeframe compared to either placebo or oral morphine [10]. Additionally, rapid-onset opioids (ROOs), particularly intranasal fentanyl spray, have been identified as a quick and effective way to manage BtCP [11]. Non-pharmacological methods can include lifestyle changes (such as limiting movement that will exacerbate pain), easing symptoms of reversible causes (such as cough suppressant medicine if a cough is causing the pain), massage, heat/cold treatments, and relaxation techniques [12, 13]. However, there is no evidence to support the efficacy of non-pharmacological treatment of BtCP [14].

Assessment and diagnosing BtCP can be difficult as universally standardized guidelines, definitions, and validated diagnostic assessment tools and processes are non-existent [7, 8]. For example, BtCP is often described as pain taking place over a short period of time, which can vary between minutes, thirty minutes, and less than an hour [8]. There are differing perspectives around the requirement to experience baseline pain, and furthermore, whether or not that pain needs to be controlled when identifying BtCP [8]. Similarly, there are differing perspectives around whether or not a numerical rating scale needs to be used to identify BtCP [8]. Identifying BtCP is not straightforward as patients often experience more than one type of BtCP, possibly requiring differing assessments [7]. The description of BtCP is not straightforward. Additionally, BtCP guidelines are not evidence-based, but rather on clinician opinion and experience [15]. The lack of international BtCP guidelines, BtCP definition consensus, and widely accepted diagnostic criteria and treatment of BtCP [15] is a source of ineffective assessment and treatment of BtCP and variability in prevalence estimates [16].

Robust BtCP research to date has focused on prevalence, effective treatments, and practice guidelines. For example, Deandrea et al. [7] conducted a systematic review of the literature to establish the prevalence of BtCP. Zeppetella et al. [10] conducted a meta-analysis to investigate the effectiveness of opioid analgesics to manage BtCP. Davies et al. [15] conducted a systematic review of national and international guidelines around BtCP management, while Suresh et al. [17] conducted a systematic review and critical appraisal of practice guidelines.

A synthesis of clinician and end-user perspectives around BtCP is non-existent. Reviews to date have synthesized quantitative evidence (surrounding prevalence [7] and treatment [10]) and health service and national and international practice BtCP guidelines [15]. Quantitative methods are valuable as they assist toward establishing the causal nature of a relationship and/or allow for the measurement of health condition prevalence [18]. However, quantitative methods do not assist in establishing meaning conveyed by people [18], which is best derived via the use of qualitative methods. The lack of a systematic synthesis of qualitative BtCP research hinders a comprehensive understanding of individual perspectives of BtCP. This, in part, contributes to a poor understanding of individual perspectives around domains which impact effective treatment [16], including definitions of BtCP, education opportunities, measures used for diagnosis, and treatment options. Furthermore, it is important that qualitative research in the area is undertaken to increase the depth of understanding and improve understanding surrounding context-specific practices.

It is important to expand the quality and quantity of research on BtCP [7, 19] with the potential to improve management and treatment. Best practice models and guidelines are likely based on findings derived from quantitative inquiry and, as a result, may not be informed by domains which lend themselves to qualitative inquiry (for example, barriers and facilitators for treatment implementation and/or social and cultural considerations). Thus, this systematic review aims to identify and synthesize the extent of qualitative research exploring health professional and patient perspectives of BtCP; it is expected that such a review will identify health professional and patient perspectives of effective treatment modalities, barriers to treatment and required education, and generate evidence-based knowledge to inform clinical practice.

## Methods

The systematic review followed the Preferred Reporting Items for Systematic Reviews and Meta-Analyses (PRISMA) approach [20]. The review protocol was registered with PROSPERO (CRD42022360350).

### Search strategy

Using the La Trobe University Online Library, on May 19, 2022, the following databases were searched for peer-reviewed literature published at any date: MEDLINE, EMBASE, and Web of Science. An evidence-based search string with BtCP-specific terms and qualitative methodology terms was developed and utilized. BtCP-specific terms were informed by Deandrea et al.’s [7] review, while qualitative method term was informed by Lippiett et al.’s [21] review. Databases were searched for sources which had ((breakthrough OR episodic OR transient OR transitory OR incident OR flare) AND (cancer OR malignant OR neoplasm OR neoplasia OR tumor OR tumour) AND pain) in the title, and (qualitative OR “case stud*” OR “focus group*” OR interview* OR phenomenolog* OR ethnograph* OR stor* OR “open ended”) in the abstract.

### Inclusion criteria

Eligible sources must have met the following criteria. Sources must have (i) been published in a peer-reviewed journal, (ii) written in English, (iii) utilized a qualitative methodology, and (iv) included the perspectives that health professionals and/or patients had surrounding BtCP. Mixed-method studies were eligible, provided they met the criteria provided and presented qualitative findings separate from any quantitative findings. Sources which did not focus specifically on BtCP and/or did not clearly describe the methodology employed were excluded.

### Screening and study selection

All sources were downloaded into an EndNote database. Initially, duplicates were removed. After, title and abstract reviews were conducted, where the suggested fields were reviewed against the inclusion criteria by a single researcher. Full texts were reviewed against the inclusion criteria by three researchers. When there was a difference of opinion surrounding the eligibility of an article, the remaining team members reviewed the source and made an inclusion conclusion.

### Synthesis and data extraction

Two researchers extracted data, and where discrepancies existed, a single member of the research team reviewed the source to rectify the discrepancy. The following information was extracted from articles and entered into a table within Microsoft Word: study aim, participants (*n*), settings, sampling, data collection, data analysis, major themes, and findings. A narrative approach (see [22]) was utilized to synthesize study findings.

### Methodological quality assessment

The methodological quality of studies was appraised using the Critical Appraisal Skills Programme (CASP) qualitative checklist [23]. The CASP checklist is a seminal qualitative checklist for health-related studies endorsed by the Cochrane Qualitative and Implementation Methods Group [24]. The checklist includes nine scored items, inclusive but not limited to the following domains: validity, bias, methodological rigor, ethical considerations, and translation. For each of the nine scored items, a response of yes is favorable and scored as a one, while a response of can’t tell, or no, is unfavorable and scored as zero. A single researcher appraised each study, and a second researcher reviewed each appraisal against the articles’ full texts. In instances where there was a discrepancy, both researchers reviewed the full text together and reached a consensus.

## Results

**Fig. 1:**
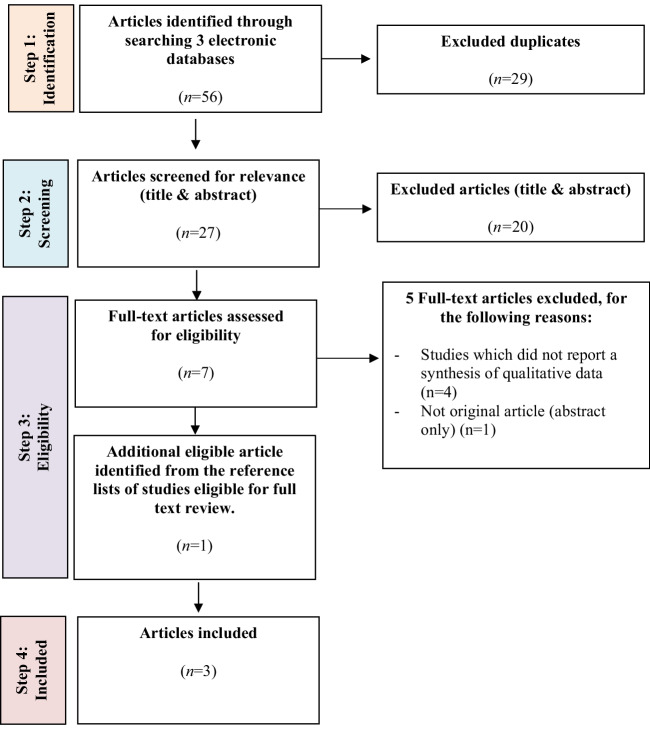
Article selection process

The search process is detailed in Fig. 1. Fifty-six sources were identified from database searches. After the exclusion of 29 duplicates, the titles and abstracts of 27 articles were reviewed against inclusion criteria. Twenty articles were excluded during the title and abstract review stage, leaving seven full-text articles for consideration. Of the seven full-text articles, five were excluded. The reference lists of the seven articles were reviewed, and a single extra source met the inclusion criteria. Thus, three sources were eligible for the review.

### Quality assessment

The CASP scoring for each study has been included in Table 1. Studies ranged from high quality [25] to moderate quality [26, 27]. Research aims, recruitment strategy, data analysis, and statement of findings criteria were addressed across the included studies. While the consideration of potential bias between researchers and participants was not. As all studies were appraised as moderate or greater, they were all considered for the review.

**Table 1:** Methodological quality assessment

Source	Was there a clear statement of the aims of the research?	Is a qualitative methodology appropriate?	Was the research design appropriate to address the aims of the research?	Was the recruitment strategy appropriate to the aims of the research?	Was the data collected in a way that addressed the research issue?	Has the relationship between researcher and participants been adequately considered?	Have ethical issues been taken into consideration?	Was the data analysis sufficiently rigorous?	Is there a clear statement of findings?	Total
Liu et al. [26]	Yes (1)	Yes (1)	Unable to tell (0)	Yes (1)	Yes (1)	No (0)	Unable to tell (0)	Yes (1)	Yes (1)	6/9 (67.8%)
Soden, Ali [25]	Yes (1)	Yes (1)	Yes (1)	Yes (1)	Yes (1)	Yes (1)	Yes (1)	Yes (1)	Yes (1)	9/9 (100%)
Webber et al. [27]	Yes (1)	Unable to tell (0)	Yes (1)	Yes (1)	Yes (1)	No (0)	Yes (1)	Yes (1)	Yes (1)	7/9 (77.8%)

### Characteristics of studies

Characteristics of studies have been provided in Table 2. Two studies investigated cancer patient perceptions of BtCP [26, 27], and one investigated nurses’ experiences of assessing and treating BtCP in palliative cancer patients [25]. Two studies were from the UK [25, 27], and one from China [26]. Study samples were small, with 9, 10, and 15 participants [25–27]. Cancer patients constituted an almost equal balance of males and females in the sample and were diagnosed with varying cancers [26, 27].

**Table 2:** Characteristics of qualitative studies on breakthrough cancer pain (BtCP)

Study	Aim	Participants (*n*)	Settings	Sampling	Data collection	Data analysis	Major themes	Quality rating	Findings
Soden et al. [25], UK	Gain a better understanding of how nurses assess and manage BtCP	Trained nurses working in specialist palliative care units (*n* = 15)	Specialist palliative care units (*n* = 5)	Semi-purposive	Semi-structured interviews conducted by consultants in palliative medicine	Thematic analysis	1. Defining BtCP 2. Assessing BtCP 3. Managing BtCP 4. Attitudes and teamwork	High	1. Lack of consistency in defining BtCP 2. Nurses adopted a holistic approach in assessing BtCP; the physical aspect of BtCP was sometimes viewed as less important than the emotional aspect 3. Nurses incorporated non-pharmacological treatments for pain management; lack of clear structure for decision-making in managing pain – predominantly patient-led 4. There is a shifting of attitudes toward pain management in palliative care; potential discord between nurses’ and doctors’ approaches to pain management
Webber et al. [27], UK	Explore the lived experience of BtCP in cancer patents	Cancer patients diagnosed with breakthrough pain (*n* = 10)	Cancer centre at 2 hospital sites	Purposive, until theme saturation occurred	In-depth interview conducted by the researcher	Content analysis methodology and thematic identification	1. Interference with ADL 2. Communication 3. Management of BtCP	Moderate	1. BtCP impacted on ADL 2. There were communication difficulties between patients and healthcare staff; most participants were unsure of the meaning of BtCP 3. Management of BtCP was via thorough assessment after referral to palliative care/pain services. Patients expressed concerns about opioid analgesia for BtCP
Liu et al. [26], China	Examine cancer patients’ perception and experience of BtCP in a remote area of China	Cancer patients diagnosed with breakthrough pain (*n* = 9)	Three wards in the oncology department at a general hospital	Non-random purposive, until theme saturation occurred	Semi-structured interviews conducted by researcher	Inductive thematic analysis	1. Suffering from BtCP 2. Hopelessness and helplessness 3. Perception of BtCP and analgesia 4. Strong as a Chinese 5. Support needed from the health care system	Moderate	1. BtCP negatively impacted patients’ ADL, sleep, thoughts, and feelings 2. Participants had a fatalistic view of cancer and BP viewed as a normal part of cancer progression; BP was often self-managed via non-pharmacological treatments or pain relief at home; there were concerns over time taken to access pain relief and staff not taking pain complaints seriously 3. Participants viewed cancer and pain as synonymous and expressed fear of addiction to opioids 4. Pain is culturally viewed as something to endure; patients were reluctant to report pain or complain; they were also hesitant to bother ‘busy’ healthcare staff 5. Causes and meaning of BtCP were poorly understood; communication between staff and patients around BtCP management was inadequate

*BtCP*, breakthrough pain; *ADL*, activities of daily living

### Themes identified through narrative synthesis

The study of patient’s perceptions of BtCP in China explored themes of suffering, hopelessness and helplessness, health care system support, views of cancer pain, and analgesia through a cultural lens [26]. Patients’ perceptions of BtCP in the UK examined the three main themes of management of breakthrough pain, breakthrough pain with activities of daily life (ADL), and communication with health care professionals [27]. Nurses’ experiences assessing and managing breakthrough pain resulted in the identification of four main themes defining BtCP, assessing BtCP, managing BtCP, and attitudes and teamwork around BtCP and patient care [25].

There were several overlapping themes across the three included studies. These themes included communication, defining BtCP, impact of BtCP, management of BtCP, perceptions of BtCP, analgesia and pain relief, and training. In relation to communication, patients clarified that there were communication difficulties with healthcare staff [26], and that communication around the management of BtCP was inadequate [27]. In relation to defining BtCP, healthcare staff indicated a lack of consistent definitions of BtCP [25], while patients were unsure of the meaning and causes of BtCP [26, 27]. In relation to the impact of BtCP, it had a negative impact on patients’ ADL, sleep, feelings, and thoughts [26, 27].

In relation to managing BtCP, nurses lacked clear guidelines for managing BtCP, so the management of BtCP was predominantly patient-led [25]. Moreover, nurses often used non-pharmacological treatment to manage BtCP [25]. There was disagreement between nurses’ and doctors’ approaches to management of BtCP [25]. Similarly, patients in a Chinese study self-managed BtCP with non-pharmacological treatments or pain relief at home [26]. Patients felt that staff did not take their complaints of pain seriously and expressed concerns over the time taken to administer pain relief [26]. Another study found that BtCP was managed after referral to a palliative care or pain service [27].

In relation to perceptions of BtCP, Chinese patients viewed BtCP as a normal part of cancer progression, and pain was culturally viewed as something to be endured [26]. On the other hand, nurses viewed the emotional aspect of BtCP as more important than the physical, incorporating a holistic approach to assessing BtCP [25]. Specific to analgesia and pain relief, patients expressed concern about the use of opioid analgesia for BtCP [27] and fear of addiction to opioids [26]. Finally, in relation to training, healthcare staff indicated the need for significant training in defining and assessing BtCP [25]. Similarly, Liu et al. [26] concluded that BtCP is poorly managed in North-western China, and staff and patients would benefit from education about BtCP.

## Discussion

This systematic review found a dearth of research using qualitative methods to explore health professional and patient perspectives of BtCP. Given research gaps identified as barriers inhibiting best practice [16], including understandings around diagnosis and management of BtCP and training and education opportunities to improve health professional practice, it was expected that the identification and synthesis of qualitative research would be of benefit. Of the three studies identified, a single study from the UK investigated nurse perspectives of BtCP and specifically focused on management strategies [25]. While two studies (one from the United Kingdom and another from China) focused on patient perspectives of BtCP [26, 27] and generally explored how BtCP impacted patients’ lives and well-being. A single consistent theme emerging from all three studies surrounded a lack of clarity of what constitutes BtCP. Additionally, the single UK study exploring the perspectives of nurses [25] and the patient-specific study from China [26] found that management strategies are largely guided by patient perceptions of what works for them.

Studies which were eligible for full-text review yet did not meet inclusion criteria – for example, due to the utilization of a qualitative design, yet not reporting qualitative findings [28, 29] [30], or the use of a structured interview where categorical and continuous data was collected [31] – have findings which are worthwhile to summarize, as they can assist in increasing our understanding of knowledge gaps which inhibit practice. A single conference abstract [32] explored communication issues between healthcare professionals and patients with BtCP. Ten patients with BtCP participated in a semi-structured interview, and themes surrounded a lack of clarity and understanding of BtCP and patient difficulties describing pain and the impact of pain on healthcare professionals. Consequently, researchers concluded that there are communication barriers between healthcare professionals and people with BtCP, which may inhibit the professional ability to diagnose and, therefore, assist in the management of BtCP. Communication difficulties exist and support patient-specific review findings confirming that communication between healthcare staff and people with BtCP is difficult [26] and perceived as inadequate [27]. In combination, the findings confirm that developing communication strategies, specifically around BtCP, are warranted. The development of robust communication strategies becomes especially important considering that the management of BtCP can be patient-led [25, 26].

Studies which were ineligible for the review generally highlight the need for further health professional education. Lopez et al. [28] investigated oncology clinician’s awareness and adherence to clinical practice guidelines for managing pain (including BtCP) in Spain. Their findings concluded that less than half of their participants participated in a pain management training program. Mercadante et al. [31] conducted structured interviews with physicians in palliative care units and hospices in Italy to establish preferred medications and routes of administration. Diversity of medication use, and particularly the use of medications which often provide an analgesic effect after the episode of pain has ceased, led the authors to conclude that clinician education opportunities to support a better understanding of effective medications are needed. Finally, quantitative findings from Soden et al.’s [30] mixed methods study confirmed that a substantial percentage of nurses within the UK (greater than 80%) indicated they would benefit from professional development and/or training. In combination, these findings support the notion that professional development and education opportunities to improve health practitioner knowledge of BtCP and effective treatments are warranted, a priority which has historically been raised [16]. Such education opportunities may also address knowledge gaps around definitions of BtCP [25–27] and respond to communication difficulties between patients and health professionals.

Current evidence on BtCP is predominantly quantitative and center around prevalence [6, 7, 33], BtCP guidelines [15, 17], and pain management [10, 13, 16, 19]. Qualitative studies of BtCP from a healthcare worker and patient perspective are lacking. Furthermore, where studies do exist, comparison is not possible due to limited existing research [31], thus inhibiting a comprehensive understanding of differing perspectives and practices based on geography. Given limited research investigating clinician perspectives of BtCP, a rich understanding informed by exploratory qualitative methods around definition, identification, best management strategies, extent of professional development, and factors promoting and inhibiting best practice remains unclear. The qualitative approach investigating “how” and “why” can complement the “what,” “where,” and “when” of quantitative approaches [34] and may provide insight into BtCP experienced by health professionals and patients.

As management of BtCP is a multidisciplinary process [16], qualitative research needs to explore the perspectives of diverse health professionals including, but not limited to, oncologists, nurses, primary care providers, and palliative care physicians located in clinical settings and the community. Furthermore, research needs to further investigate poorly understood domains inhibiting practice [16] surrounding defining BtCP, management strategies (including factors promoting and inhibiting management), and training and education required. Such research would benefit from consensus-building methods to establish, for example, an international universally agreed definition of BtCP. While largely quantitative methods underpinned by descriptive statistics have been useful to develop an understanding of clinician competencies and preferred management strategies [28], and confirmation as to whether or not training is required [30], qualitative methods which explore the experiences and perspectives of health professionals will be an important step forward and assist in developing a comprehensive understanding of the health professional experiences and necessary training, and particularly a nuanced understanding based on differing professions and locations of practice.

To date, consumer involvement in palliative care practice and research is lacking. Palliative care service models are generally informed by practitioners’ perceptions of patients’ needs [35, 36] rather than the needs identified by patients. A systematic review which aimed to synthesize the extent of research focusing on consumer and carer leadership in palliative care identified eleven sources; across these sources, the extent of consumer involvement was unclear [36]. A 2020 systematic review synthesized research clarifying palliative care research priorities [37]; of the ten sources identified, consumer and caregiver priorities were largely absent. Findings from this review confirmed that aspects of BtCP management are patient-led [25, 26], suggesting that exploring the patient perspective around BtCP is especially important. Notwithstanding the need for qualitative research involving health professionals, it is also important that further research involves end users, as their perspective can ensure BtCP management practice is also patient-centered.

### Limitations

This review has limitations which are important to consider. The review identified and synthesized research from three seminal health research databases – MEDLINE, EMBASE, and Web of Science. While these three databases are comprehensive, it is possible that sources existing outside of the suggested databases were not included. Furthermore, it is possible that sources which did not include terms included within the search string were not included. However, as an evidence-based search string with BtCP-specific terms and qualitative methodology terms were developed and used (informed by Deandrea et al. [7] and Lippiett et al. [21]), the authors expect that the exclusion of articles due to the search string remains unlikely.

## Conclusion

BtCP is prevalent among people who experience cancer, with an impact on psychological and emotional health and activities of daily living. BtCP can be difficult to identify, manage, and treat, and this is in part due to a poor understanding around health professional competencies, areas requiring training and education, management strategies, and patient perspectives of these management strategies. Qualitative inquiry is warranted, and it is expected that qualitative findings can inform clinical guidelines used in the practice of health professionals surrounding their work toward the management of BtCP. A subsequent task becomes ensuring that health professionals are aware of updated clinical guidelines and are well supported in implementing these guidelines within practice.

## Data Availability

Peer-reviewed articles which met review criteria can be accessed via relevant publishers.
